# Luxury Vegetative Nitrogen Uptake in Maize Buffers Grain Yield Under Post-silking Water and Nitrogen Stress: A Mechanistic Understanding

**DOI:** 10.3389/fpls.2019.00318

**Published:** 2019-03-26

**Authors:** Joshua Nasielski, Hugh Earl, Bill Deen

**Affiliations:** Department of Plant Agriculture, University of Guelph, Guelph, ON, Canada

**Keywords:** grain nitrogen sources, maize, nitrogen remobilization, nitrogen stress, post-silking nitrogen uptake, water stress, yield

## Abstract

During vegetative growth maize can accumulate luxury nitrogen (N) in excess of what is required for biomass accumulation. When post-silking N uptake is restricted, this luxury N may mitigate N stress by acting as an N reserve that buffers grain yield and maintains plant function. The objective of this study was to determine if and how luxury accumulation of N prior to silking can buffer yield against post-silking N and/or water stress in maize. In a greenhouse experiment, maize was grown in high (N^veg^) and low (n^veg^) N conditions during vegetative growth. The n^veg^ treatment did not affect biomass accumulation or leaf area by silking but did accumulate less total N compared to the N^veg^ treatment. The N^veg^ treatment generated a reserve of 1.1 g N plant^-1^. Plants in both treatments were then subjected to water and/or N stress after silking. ^15^N isotope tracers were delivered during either vegetative or reproductive growth to measure N remobilization and the partitioning of post-silking N uptake with and without a luxury N reserve. Under post-silking N and/or water stress, yield was consistently greater in N^veg^ compared to n^veg^ due to a reduction in kernel abortion. The N^veg^ treatment resulted in greater kernel numbers and increased N remobilization to meet grain N demand under post-silking N stress. Luxury N uptake at silking also improved leaf area longevity in N^veg^ plants compared to n^veg^ under post-silking N stress, leading to greater biomass production. While post-silking N uptake was similar across N^veg^ and n^veg^, N^veg^ plants partitioned a greater proportion of post-silking N to vegetative organs, which may have assisted with the maintenance of leaf function and root N uptake capacity. These results indicate that N uptake at silking in excess of vegetative growth requirements can minimize the effect of N and/or water stress during grain-fill.

## Introduction

In maize (*Zea mays* L.) as in other globally important cereals, nitrogen (N) taken up during vegetative growth before silking is later remobilized to the ear to support kernel formation. Roughly 45–65% of maize grain N at maturity is derived from N remobilization, while N taken up during reproductive growth supplies the rest (Gallais et al., 2007; Ciampitti and Vyn, 2012; Ning et al., 2017). But under abiotic stresses the typical patterns of N remobilization and partitioning are disrupted (Ciampitti and Vyn, 2013; de Oliveira Silva et al., 2017). Notably, when whole-season or post-silking N availability is low, grain N will rely increasingly on remobilized N (Friedrich and Schrader, 1979; Ta and Weiland, 1992), possibly acting as a compensatory mechanism to support kernel N demand when post-silking N uptake is low (Ciampitti and Vyn, 2012; Mueller et al., 2017). This phenomenon is also observed in wheat (Palta et al., 1994). However, an increase in N remobilization under post-silking N stress can lower yield by reducing leaf and root function, thereby decreasing assimilate production and nutrient capture earlier than normal (Triboi and Triboi-Blondel, 2002). While maize can utilize previously acquired N for grain development, it relies heavily on concurrent assimilate supply to meet grain assimilate demand. This leads to the “yield dilemma” of N remobilization (Masclaux-Daubresse et al., 2010), whereby high rates of N remobilization are tied to a decrease in yield due to leaf senescence reducing assimilate production during grain-fill. As N remobilization increases in maize, post-silking N uptake is normally reduced as well (Gallais and Coque, 2005; Ciampitti and Vyn, 2013). Presumably this is because N remobilization also reduces assimilate supply to roots (Muchow, 1988; Ta and Weiland, 1992; Pan et al., 1995; Liedgens et al., 2000), but also perhaps because N remobilization reduces the demand for post-silking N (Mueller and Vyn, 2018).

In well-fertilized farm fields, the risk of post-silking N stress is largely due to excessive rainfall events that lead to large field-scale N losses via leaching or denitrification (Eagle et al., 2017), or water deficits, which can limit N availability (Gonzalez-Dugo et al., 2010). Under droughty conditions maize roots can access water in deeper soil layers, but N is concentrated in the topsoil and arrives to the root surface via mass flow or diffusion, assisted by transpiration (Kim et al., 2008; McMurtrie and Näsholm, 2018). Water stress reduced biomass accumulation rates and yield, particularly when it is experienced around silking (Cakir, 2004), and because water stress reduces maize growth, it also lowers the N uptake needs of the plant (Gonzalez-Dugo et al., 2010). However, the reduction in maize N uptake that is commonly observed under water limitation (e.g., Teixeira et al., 2014) is not solely attributable to a reduction in maize growth, but also to a direct reduction in the accessibility of soil N for crop uptake (Lemaire et al., 1996; Buljovcic and Engels, 2001).

Plénet and Lemaire (2000), as well as Ciampitti and Vyn (2012), hypothesized that the accumulation of luxury N – N taken up during vegetative growth that is not used for biomass production but instead incorporated into storage pools – may be able to buffer against N stresses experienced during grain-fill. In the former study, irrigated maize was found to benefit from luxury pre-silking N uptake beyond that required for maximum biomass accumulation, concluding that a luxury N pool of ∼30 kg N ha^-1^ by silking may be beneficial (Plénet and Lemaire, 2000). The authors concluded that this reserve of vegetative N buffers grain yield since soil N availability during grain-fill is normally insufficient to support N demand (Plénet and Lemaire, 2000). But the possible mechanism(s) underlying this response to luxury N uptake, such as the quantification of grain N sources or changes in canopy longevity, were not explored. In the latter study, a synthesis-analysis, the authors observed that grain yield was strongly correlated to N uptake at silking (Ciampitti and Vyn, 2012). Based on this relationship the authors suggested that, as a pathway to yield improvement, maize breeding efforts should focus on increasing pre-silking N uptake and forming an N reserve. However, no assessment was made as to the importance of a pre-silking N reserve under post-silking N or water stress (Ciampitti and Vyn, 2012). Both studies reason that, by minimizing dependence on post-silking N uptake, a reserve of luxury N accumulated by silking can allow the plant to meet grain N demand when post-silking N uptake is low.

Several researchers have used ^15^N isotope tracing to quantify maize response to post-silking N stress. In a greenhouse experiment, Friedrich and Schrader (1979) labelled maize with ^15^N during vegetative growth and then imposed N stress after silking. N remobilization from leaves, stems and roots was enhanced under post-silking N stress relative to control. In a field study using ^15^N multi-stage pulse labelling, de Oliveira Silva et al. (2017) quantified differences in post-silking N allocation to plant organs at discreet time periods during grain-fill. When no N was applied, a much greater proportion of post-silking N was allocated to developing ears, at the expense of leaves, relative to a moderately fertilized control. In a greenhouse experiment, Paponov and Engels (2005) applied ^15^N at the start of grain-fill to measure the partitioning of post-silking N under high and low N supply. A greater proportion of post-silking N uptake was allocated to the grain under low N and they noted that under N stress, N remobilization from leaves was enhanced relative to high N conditions (Paponov and Engels, 2005). Together these results demonstrate that under post-silking N stress, maize N metabolism changes to ensure that ear or kernel N supply is conserved at the expense of the N status of vegetative organs. As such they are in concordance with the idea that a reserve of luxury pre-silking N can buffer yield under post-silking N stress by allowing greater N remobilization to the grain without impinging on leaf or root function.

The objectives of this study were to determine: (i) if maize has the capacity to accumulate N in excess of that required for biomass production (luxury N uptake), (ii) quantify the contribution of pre-silking N remobilization and post-silking N uptake as sources of grain N, and (iii) measure biomass accumulation, leaf area, leaf carbon exchange rates (CER), grain yield and yield components to determine the potential value of luxury N to buffer against post-silking N and water stress.

## Materials and Methods

### Greenhouse Experiment Setup and Design

The experiment was performed in the Crop Science Greenhouse at the University of Guelph, ON, Canada. Seeds of the maize hybrid DKC39-97 (Dekalb (Monsanto); Winnipeg, MB, Canada) were sown individually in 19-L plastic pots, filled with Turface MVP^®^ (Profile Products LLC; Buffalo Grove, IL, United States), an inert calcined clay widely used as growing medium for maize (Goron et al., 2015). All pots were free-draining due to holes on the bottom, with a mesh screen ensuring roots did not exit the pots. A constant temperature regime of 27°C day/21°C night was used throughout the experiment, while high pressure sodium and metal halide lamps provided supplemental lighting on a 16-h photoperiod starting at dawn.

A nested factor design was used in the experiment with two N supply treatments (high and low N), imposed during vegetative growth under well-watered conditions. Five days after R1 (silking) (Abendroth et al., 2011) plants were placed into one of four treatments consisting of two N supply levels (high and low N) and two levels of water supply (well-watered and water stress) applied in combination. The experiment was setup as a completely randomized block design, with six blocks (i.e., replicates) each containing all eight treatments. Treatments were applied to individual plants, and plant pots were rotated within the blocks every 7 to 10 days to avoid border effects.^15^N isotope tracers were delivered to plants in four of the six blocks, with plants labelled once during either vegetative or reproductive growth. The other two blocks were treated identically except no ^15^N tracers were added. Blocks with ^15^N contained 24 plants while blocks not receiving ^15^N contained 16 plants. Total time from emergence to maturity was 90 days. The experiment was conducted in two separate greenhouses, each greenhouse containing three blocks, two blocks with ^15^N labelling and one block without ^15^N labelling.

#### Nitrogen and Water Supply Treatments

Final concentration of N was 20 mM in the high N solution and 5 mM in the low N solution. The high N and low N treatments were based on pre-experiments done in preparation for the present study. The low N treatment (5 mM) was selected because it could achieve similar biomass and leaf area at silking compared to 20 mM, but 20 mM generated much greater N uptake. Pre-experiments found that the N limitation at 5 mM was relatively mild, but sufficient to reduce grain yield relative to 20 mM. While several researchers have imposed N stress by withholding fertilizer N completely (e.g., Subedi and Ma, 2005), the growing medium those experiments used contained a proportion of field soil that could provide N for plant uptake via mineralization. Turface does not provide any N for plant uptake (Goron et al., 2015).

Each fertilizer treatment was delivered on a separate, parallel, solenoid system with automatic fertilizer injectors (Superdos; Dosmatic U.S.A., Inc.) and fertilizer solution was delivered to the pot surface with a spray stick (Acu-Spray Stick; Jain Irrigation). The nutrient mixture, based on the recipe of Echarte et al. (2008), was made by dissolving into 80 L of distilled water: 0.329 L H_3_PO_4_ (750 g kg^-1^), 0.600 kg KHCO_3_, 0.320 kg Ca(NO_3_)_2_, 0.64 kg MgSO_4_, and 0.048 kg of chelated micronutrient mix (Plant Products Co., Brampton, ON, Canada). In the high N nutrient solution 1.125 kg NH_4_–NO_3_ was added, and in the low N nutrient solution, 0.165 kg NH_4_–NO_3._ The mixture was diluted (20 times) during irrigation with distilled water. In addition to the nutrient mixture, distilled water irrigation was provided individually to each pot using an irrigation system that was parallel to the fertigation system described above.

Volumetric water content was monitored via 30 soil moisture sensors (EC-5; Decagon Devices, Pullman, WA, United States), with a minimum of 1 sensor in each treatment per block. All sensors were fitted with a custom calibration for use in Turface and outputs were coupled with a moisture characteristic curve for Turface calculated with the pressure plate technique (Premalal, 1997). Well-watered plants were irrigated to keep volumetric soil moisture between 35 and 40% (targeting –35 millibar), corresponding to a plant available water content between 80 and 90% of maximum. Pots in the water stress treatment imposed after silking were irrigated to keep volumetric soil moisture between 25 and 30% (targeting –500 millibar), equivalent to 57–68% of maximum plant available water content. These levels of water supply were selected based on a previous experiment that measured maize response to water supply in Turface. The level of water stress was chosen to be severe enough to reduce yield relative to well-watered plants without provoking terminal drought. Throughout the experiment, fertigation and irrigation water combined never exceeded the water holding capacity of the pots except at silking when pots were being transitioned from vegetative to reproductive treatments.

#### Treatment Imposition and ^15^N Labelling During Vegetative or Reproductive Growth

Nineteen days after plant emergence, when four fully emerged collared leaves were present (V4 growth stage; Abendroth et al., 2011) both high N (N^veg^) and low N (n^veg^) were imposed under well-watered conditions. Each plant was monitored for the first appearance of silks. Five days after silk appearance the remaining plants were irrigated with distilled water to remove residual N in the growing medium and placed into one of the four post-silking treatments that lasted until maturity. These treatments were: (1) high N, well-watered (NW^rep^), (2) low N, well-watered (nW^rep^), (3) high N, water stress (Nw^rep^), or (4) low N, water stress (nw^rep^).

In the four blocks receiving ^15^N, plants were labelled once, either during vegetative growth or after silking during reproductive growth. During vegetative growth, a total application of 40 mg of ^15^N was applied to label plants in both N^veg^ and n^veg^. Four times over a labelling period of 15 days, 10 mg of ^15^N was delivered in a syringe by dissolving 680 mg of K^15^NO_3_ at 10% ^15^N abundance (purchased as 10% K^15^NO_3_ from Sigma-Aldrich; St. Louis, MO, United States) in 100 mL of water. The daily irrigation regime ensured that the applied N infiltrated into the root zone. At each ^15^N application date, all plants not receiving ^15^N during vegetative growth were given the same amount of unlabelled KNO_3_ in 100 mL of water to ensure equivalent amounts of water and KNO_3_ were provided to all plants. Plants labelled during reproductive growth received 90 mg of ^15^N. Nine times over a period of 24 days, beginning 8 days after silking, 10 mg of ^15^N was delivered to the base of each plant in the same process used during vegetative labelling. Plants that were not being labelled during reproductive growth were provided with equivalent amounts of water and KNO_3_ at each ^15^N application date to avoid confounding effects. In all eight treatment combinations, when plants were harvested at maturity, one plant was labelled during vegetative growth and one plant was labelled during reproductive growth per block.

### Irrigating Pots at Silking to Remove ^15^N Applied During Vegetative Growth to Improve Estimates of N Remobilization to the Grain

To improve estimates of pre-silking N remobilization to the grain, ^15^N was removed from each pot at silking by irrigating pots for 30 min at a flow rate of 18 L min^-1^ and allowing excess water to continually drain from the bottom holes of the pot. Two studies were conducted to test the efficacy of this procedure in leaching residual ^15^N and preventing post-silking ^15^N uptake. To test the leachability of Turface MVP^®^, 2.25 g of N (in the form of dissolved KNO_3_) was added to 19-L pots filled with Turface MVP^®^ without exceeding the water holding capacity of the pots. After resting for 1 day, pots were irrigated for 30 min or not at all (*n* = 4) and residual N in the Turface growing medium was measured via colorimetric analysis analogous to soil sampling for N content. Compared to the non-irrigated pots, Turface N content of the irrigated pots was 99% lower, indicating that most of the 2.25 g N added to each pot was leached away following the 30 min irrigation (Supplementary Table S1). As a second experiment, 40 mg of ^15^N was delivered via syringe to four 19-L pots filled with Turface MVP^®^ by dissolving 2.25 g of K^15^NO_3_^-^ at 10% ^15^N abundance in 400 mL of water. After 3 days, pots were irrigated for 30 min each (flow rate = 18 L min^-1^). Each pot was immediately sown with a hybrid maize seed DKC39-97 (Dekalb (Monsanto); Winnipeg, MB, Canada) and grown until V13 (Abendroth et al., 2011) in non-stress (high N and well-watered) conditions. At V13 the whole plant was harvested, ground and the Delta^-15^N (δ^15^N ‰) was determined to see if enrichment of plant tissue above natural abundance occurred. The δ^15^N ‰ is a measure of the level of ^15^N enrichment or depletion in a sample relative to natural abundance, with δ^15^N equal to zero at the international isotope standard of atmospheric N_2_ (0.3663% ^15^N) (Mariotti et al., 1981). The δ^15^N (‰) of these plants varied from –1.59 to –3.88 (0.3649 to 0.3657% ^15^N), indicating that the isotopic ratio was slightly below natural abundance and no excess ^15^N uptake occurred after the irrigation procedure (Supplementary Table S2).

### Measurements

At silking four plants in N^veg^ and four plants in n^veg^ were harvested in each block. Plant roots were first rinsed in distilled water and plants were dissected into 4 organ groups: leaves, stalk, roots and reproductive organs (cob, silks, tassel and husk). All plant parts were dried at 80°C in an oven for 48 h and weighed. On half of the harvested plants, the organs (leaves, stalk, roots, reproductive organs) were ground into a homogenous powder, and a 10-g subsample was used to determine total N content using dry combustion (Dumas method). Leaf area was measured for each harvested plant by passing leaves through a leaf area meter (LI-3100c, LI-COR, Lincoln, NE, United States). On a subset of leaves, leaf length and width at the widest point were also measured with a ruler prior to the leaf area meter measurement, and then later dried in an oven at 80°C for 48 h and weighed individually. These measurements were used to parameterize two regressions, one regressing leaf length and width (as measured by a ruler) to leaf area (cm^2^), and another regressing leaf weight to leaf area (cm^2^) (described in the Supplementary Material). These regressions were used to calculate leaf area during grain-fill. Potential kernel number was measured following Echarte and Tollenaar (2006), with spikelets at the tip of the cob, where kernel rows became non-uniform, not counted.

Leaf area was measured non-destructively at tasseling on all plants, by which time leaf emergence was complete. Length and width (at the widest point) of each individual leaf was measured with a ruler. Results from the destructive harvest at silking enabled ruler-based measurements of leaf length and width to be converted to leaf area. Leaf senescence during reproductive growth was measured one, two and three weeks after silking. Fully senesced leaves or parts of leaves which were senesced, identified visually as lacking green colour, and by touch as being brittle (Wolfe et al., 1988) were cut off from green areas using scissors. Senesced leaves or leaf fragments were dried for 48 h at 80°C and then weighed. Leaf area (cm^2^) was estimated based on dried leaf weight (g) using a regression parameterized using leaf data collected at silking (described in the Supplementary Material).

Leaf CER was measured with a LI-6400XT (LI-COR, Lincoln, NE, United States) at 2000 μmol m^-2^ s^-1^ photosynthetic photon flux density at the leaf surface, maintaining a CO_2_ concentration of 350 ppm and a leaf temperature of 30°C within the sample chamber. Measurements were taken on three leaves per plant: (i) the second leaf below the ear leaf, (ii) the ear leaf, and (iii) the second leaf above the ear leaf. Leaf CER was measured at mid-day, in a 6 cm^2^ area near the middle of the leaf that did not include the midrib. Measurements were made three times during the experiment: (i) at the beginning of silking, before the imposition of the reproductive treatments, (ii) 21 days after silking, and (iii) 34 days after silking.

At physiological maturity 80 plants were harvested, 16 from each of the four blocks receiving ^15^N and 8 from each of the two blocks not receiving ^15^N. Roots were washed immediately in distilled water, and all plants were dissected and organs placed into five groups: green leaves (or parts of green leaves), senesced leaves (or parts of senesced leaves), stalk, roots, grain, and non-grain reproductive organs (cob, husk and tassel). Plant parts were then dried at 80°C in an oven for 48 h and weighed. Kernel number was determined in a computerized seed counter (ESC^-1^; Agriculex, Guelph, ON) which allowed for average kernel weight to be calculated. Plant samples were analyzed for N concentration and ^15^N/^14^N ratio by dry combustion in an elemental analyzer (CHNS-O 1108; Carlo Erba, Italy) coupled to a continuous-flow stable isotope ratio mass spectrometer (DeltaplusXL; Thermo Finnigan, Germany) at the University of Waterloo Environmental Isotopes Laboratory in Waterloo, Canada.

### ^15^N Calculations

Calculations of N uptake, remobilization and partitioning were based on Paponov and Engels (2005), Gallais et al. (2006, 2007) and Ning et al. (2017). They were made assuming: (1) no isotopic discrimination between ^14^N and ^15^N within the maize plant, (2) no isotopic discrimination in reactions involved in N metabolism, (3) no N losses from the plant and (4) that ^15^N was uniformly distributed in the growing medium surrounding the roots (Gallais et al., 2006, 2007). ^15^N occurs naturally in maize tissue at approximately natural abundance (0.3663% ^15^N), and ^15^N accumulating in excess of this amount in a plant organ is assumed to come from ^15^N fertilizer. Total plant ^15^N derived from fertilizer could be calculated and the sum of ^15^N in each plant organ in excess of natural abundance. Because all plants labelled with ^15^N during vegetative growth were irrigated for 30 min at silking, it was assumed that that no residual ^15^N applied during vegetative growth was taken up after silking. The equations used in ^15^N calculations of N remobilization normally use a correction factor to account for this residual post-silking ^15^N uptake (e.g., Ning et al., 2017), but no correction factors are used here. In this study, the natural abundance of unlabelled maize grown in the greenhouse was determined empirically (*n* = 5) and a value close to the international standard of atmospheric N_2_ was used (range: 0.3733 ± 0.0031 %^15^N).

#### Calculation of Vegetative N Partitioning and Remobilization at Maturity

For every plant part sampled (e.g., roots, stalk, leaves), the amount of ^15^N derived from labelled fertilizer (i.e., the amount of ^15^N in excess over natural abundance), was calculated as:

(1) $$q_{\left(x\right)}=\left(A_{\left(x\right)}-\;A_{O\left(x\right)}\right)\times \;Q_{x}$$

where, *q_(x)_* equals the amount of ^15^N derived from labelled fertilizer (i.e., ^15^N% excess for part x), *A_(x)_* equals ^15^N abundance for organ *x, A_O_* equals 0.3733% ^15^N (i.e., natural abundance of ^15^N in maize for this experiment) and *Q_x_* is the total N content of part *x*.

For plants labelled prior to silking, the amount of ^15^N excess found in the grain *q_(grain)_* was assumed to come from ^15^N excess originating from pre-silking N uptake of labelled fertilizer that was then remobilized. The percent of pre-silking N remobilized (*rem%*) is thus equal to:

(2) $$rem\%=\;\frac{q_{\left(grain\right)}}{q_{\left(wpm\right)}}\;\;\times 100$$

where, *q_grain_* is the amount of excess ^15^N in the grain, and *q_(wpm)_* is the amount of excess ^15^N in the whole plant at maturity. Consequently, the amount of pre-silking N remobilized to the grain at maturity (*Q_rem(grain)_*) is equal to:

(3) $$Q_{rem\left(grain\right)}=\;Q_{\left(wps\right)}\;\times rem\%$$

where, *Q_(wps)_* is equal to the total N content of the whole plant at silking. The percentage of grain N derived from remobilization (*Grain_rem_*) is equal to:

(4) $$Grain_{rem}=\;\frac{Q_{rem\left(grain\right)}}{Q_{\left(grain\right)}}\;\times 100$$

where, *Q_(grain)_* is the N content of the grain.

#### Calculations of Post-silking N Uptake and Partitioning

For plants labelled during reproductive growth the percentage of post-silking N uptake partitioned to the grain (*Post%_(grain)_*) is equal to:

(5) $$Post{\%}_{\left(grain\right)}=\;\frac{q_{\left(grain\right)}}{q_{\left(wpm\right)}}\;\;\times 100$$

Note that Eq. 5 is analogous to Eq. 2, used when plants were labelled during vegetative growth. It follows that the total amount of grain N originating from post-silking N uptake (*Q_post(grain)_*) is equal too:

(6) $$Q_{post\left(grain\right)}=\left(\;Q_{\left(wpm\right)}-\;Q_{\left(wps\right)}\right)\times Post{\%}_{\left(grain\right)}$$

Note that Eqs 5 and 6 are generalizable to any plant organ. *Q*_(_*_wps_*_)_ was the average N uptake for either vegetative treatments (N^veg^ and n^veg^) (*n* = 12). *Q*_(_*_wpm_*_)_ was measured individually for each plant. For plants labelled during reproductive growth, the percent of grain N derived from remobilization (*Grain_rem_*):

(7) $$Grain_{rem}=\left(1-\frac{Q_{post\left(grain\right)}}{Q_{\left(grain\right)}}\right)\;\times 100$$

### Statistical Analysis

All statistical analyses were performed in R v.3.5.0 (R Core Development Team; Vienna, Austria) using a mixed effects model (lme function; nlme R package) with vegetative and reproductive treatments as fixed factors; random factors were replicate and greenhouse. The experiment was set up as a nested factor design (Eq. 8), with eight treatments in total (4 reproductive treatments and 2 vegetative treatments), and all statistical analyses were computed with vegetative treatments nested within reproductive treatments via the lsmeans function (lsmeans R package). Proportional data were analyzed using a beta regression (glmmTMB function; R package glmmTMB) and count data using a Poisson regression (glmer function; R package lme4). Green leaf area per plant during grain-fill was analyzed with a repeated measures ANOVA (lmer function; lme4 R package). Multiple means comparisons were performed with a Tukey’s test. For all statistical tests conducted, the type I error rate was set at 0.05. After fitting, all models were assessed for normally distributed residuals and homogenous error variance across factor levels; when residual non-normality or heterogeneity among factor levels was apparent the model covariance structure was adjusted to a heterogenous error model.

(8) $$Y_{ijkl}=\mu +\;\beta_{i}+\;\;\beta_{j\left(i\right)}+\;\alpha_{k}+\;\alpha_{l\left(k\right)}+\;\varepsilon_{ijkl}$$

where, μ is the overall mean, β*_i_* is the fixed effect of the reproductive treatment *i*, β*_j(i)_* is the fixed effect of the vegetative treatment *j* nested within reproductive treatment *i*,α*_k_* is the random effect of greenhouse *k*, α*_l(k)_* is the random effect of block *l* within greenhouse *k* and ε*_ijkl_* is the random error.

## Results

### Luxury N Uptake Was Observed in the N^veg^ Treatment Relative to n^veg^ at Silking

**FIGURE 1:**
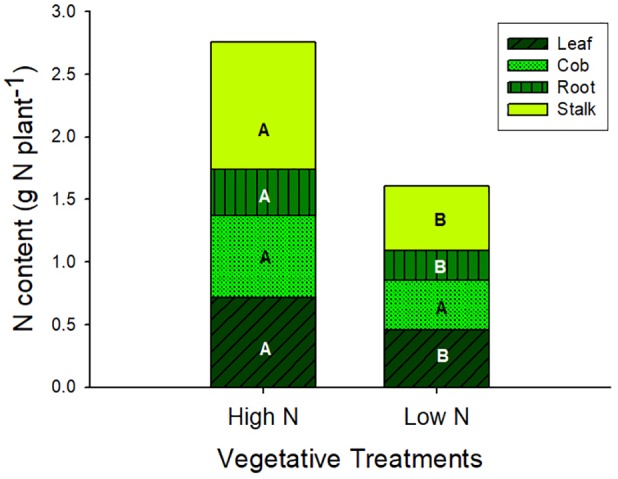
Plant N content at silking partitioned across vegetative organ in the vegetative treatments (mean ± S.E.). Different letters indicate significant differences in organ N content across the high N (N^veg^) and low N (n^veg^) vegetative treatments at *p* < 0.05 (*n* = 6).

At silking total biomass and leaf area were statistically similar across n^veg^ and N^veg^, although total N content was greater in N^veg^ (Table 1). Leaf CER was the same across N^veg^ and n^veg^ treatments (mean = 24.2 μmol m^2^ s^-1^) at all three sampled leaf positions (Supplementary Figure S1). This indicates that luxury N was accumulated during vegetative growth in the N^veg^ treatment. This luxury N accumulated primarily in the stalk and leaves (Figure 1). There was no difference in silking date (data not shown). N^veg^ had a greater spikelet count compared to n^veg^ (805 vs. 656 spikelets plant^-1^) indicating that the increased N uptake in N^veg^ stimulated greater spikelet formation (Table 1).

**Table 1:** Plant parameters (mean ± S.E.) at silking across the high N (N^veg^) and low N (n^veg^) treatments.

Vegetative Treatment	Biomass (g plant^-1^)	Leaf area (cm^2^ plant^-1^)	Spikelet count (spikelet plant^-1^)	N uptake (g N plant^-1^)
N^veg^	176.8 ± 12.1 a	3538 ± 157 a	805 ± 25 a	2.76 ± 0.30 a
n^veg^	156.4 ± 8.4 a	3231 ± 112 a	656 ± 21 b	1.61 ± 0.19 b


*Means within a column for each parameter followed by different letters (a–b) are significantly different at *p* < 0.05 (*n* = 6).*

### Grain N Sources and Post-silking N Partitioning in Vegetative and Reproductive Treatments

Based on ^15^N measurements post-silking water and/or N stress increased the percent of grain N derived from remobilization in both the N^veg^ and n^veg^ treatment (Table 2). In N^veg^, the proportion of grain N derived from remobilized increased from 61.9% under NW^rep^ to an average of 85.9% in the reproductive stress treatments (nW^rep^, Nw^rep^ and nw^rep^) (Table 2). Under n^veg^ these values were 45.4 and 65.8% for NW^rep^ and the stress treatments, respectively. Without a reserve of luxury N, the amount of N remobilized to the grain in n^veg^ under post-silking stress was 0.77 g N plant^-1^, lower than the amount of N remobilized in N^veg^ (1.57 g N plant^-1^) but as a proportion of total pre-silking N, slightly greater (62.5% vs. 55.6%) (Supplementary Figure S2) according to ^15^N calculations. Overall, under post-silking water and/or N stress, a greater amount of pre-silking N was remobilized in N^veg^ compared to n^veg^, and this remobilized N comprised a larger proportion of grain N (Table 2).

**Table 2:** Effect of the vegetative treatments (N^veg^ and n^veg^) on the proportion of grain N derived from remobilization and amount of pre-silking N remobilized to the grain as measured by the ^15^N method (mean ± S.E) across all four reproductive treatments (NW^rep^, nW^rep^, Nw^rep^, nw^rep^) at maturity.

Reproductive Treatments	Vegetative Treatments	Proportion of grain N derived from N remobilization (%)	Grams of pre-silking N remobilized to the grain (g N plant^-1^)
High N + Well watered (NW^rep^)	N^veg^	61.9 ± 8.6 a	1.59 ± 0.24 a


	n^veg^	45.4 ± 4.0 a	0.91 ± 0.11 b
Low N + Well watered (nW^rep^)	N^veg^	82.0 ± 5.4 a	1.67 ± 0.08 a


	n^veg^	64.0 ± 8.0 b	0.82 ± 0.11 b
High N + Water Stress (Nw^rep^)	N^veg^	87.2 ± 4.7 a	1.63 ± 0.08 a


	n^veg^	72.1 ± 3.4 b	0.77 ± 0.11 b
Low N + Water Stress (nw^rep^)	N^veg^	88.2 ± 2.9 a	1.42 ± 0.07 a


	n^veg^	61.2 ± 10 b	0.59 ± 0.12 b


*Within reproductive treatments, vegetative treatment means for each parameter followed by different letters (a–b) are significantly different at *p* < 0.05 (*n* = 4). Vegetative treatments means are always compared within reproductive treatments.*

Post-silking N and/or water stress treatments reduced post-silking N uptake relative to NW^rep^ in both vegetative treatments (Table 3). Post-silking N uptake was similar for N^veg^ and n^veg^ in all four reproductive treatments (Table 3), indicating that differences in N remobilization and pre-silking N uptake did not affect post-silking N accumulation. However, the allocation of post-silking N across plant organs differed between N^veg^ and n^veg^ according to ^15^N measurements. Notably, under low N supply (nW^rep^ and nw^rep^) n^veg^ plants partitioned more post-silking N to the grain relative to N^veg^ (79.4% vs. 31.8%) (Table 3). Aside from the grain, by maturity the stalk was a major sink for post-silking N in N^veg^, accounting for 23.8% of post-silking N uptake (Table 3). Under n^veg^, the stalk was a much smaller sink for post-silking N except when adequate N and water supply was supplied (NW^rep^). The roots were also a major sink of post-silking N, especially under water stress. Under water stress (Nw^rep^ and nw^rep^) plants in N^veg^ partitioned more post-silking N to the roots relative to n^veg^ (46.3% vs. 26.7%), and overall results indicate that under post-silking water stress the roots are a major sink for post-silking N (Table 3). The cob and leaves (both senesced leaves and green leaves) each accounted for less than 5% of post-silking N in all treatments, except for n^veg^-NW^rep^, where post-silking N accumulated in the cob and leaves to a much greater extent (Table 3).

**Table 3:** Effect of the vegetative treatments (N^veg^ and n^veg^) on post-silking N uptake and partitioning of post-silking N uptake (mean ± S.E) across all four reproductive treatments (NW^rep^, nW^rep^, Nw^rep^, nw^rep^) at maturity.

Reproductive Treatments	Vegetative Treatments	Post-silking N uptake (g N plant^-1^)	Proportion of reproductive N allocated to the grain (%)	Proportion of reproductive N allocated to the cob/husk (%)	Proportion of reproductive N allocated to the stalk (%)	Proportion of reproductive N allocated to the roots (%)	Proportion of reproductive N allocated to green leaves (%)	Proportion of reproductive N allocated to senesced leaves (%)
High N + Well Watered (NW^rep^)	N^veg^	1.35 ± 0.23 a	64.6 ± 5.8 a	1.7 ± 0.4 a	20.3 ± 4.8 a	11.5 ± 2.1 a	0.3 ± 0.1 a	2.1 ± 0.1 a
	n^veg^	1.75 ± 0.31 a	32.7 ± 9.2 b	14.3 ± 6.3 b	30.9 ± 6.7 a	14.7 ± 2.9 a	5.1 ± 2.2 b	2.2 ± 0.4 a
Low N + Well Watered (nW^rep^)	N^veg^	0.58 ± 0.15 a	69.0 ± 4.0 a	1.8 ± 0.3 a	19.1 ± 3.4 a	7.1 ± 1.4 a	1.5 ± 0.9 a	1.5 ± 0.4 a


	n^veg^	0.50 ± 0.15 a	86.4 ± 2.9 b	3.0 ± 0.3 a	4.6 ± 1.0 b	4.0 ± 2.1 a	0.6 ± 0.6 a	1.5 ± 0.3 a
High N + Water Stress (Nw^rep^)	N^veg^	0.61 ± 0.26 a	25.6 ± 4.0 a	1.7 ± 0.2 a	35.7 ± 6.8 a	33.9 ± 6.3 a	1.9 ± 1.2 a	1.2 ± 0.9 a


	n^veg^	0.69 ± 0.14 a	30.4 ± 2.9 a	9.3 ± 4.0 b	19.8 ± 8.2 b	37.3 ± 1.4 b	2.5 ± 1.8 a	0.6 ± 0.2 a
Low N + Water Stress (nw^rep^)	N^veg^	0.67 ± 0.12 a	17.0 ± 3.9 a	2.4 ± 0.9 a	20.3 ± 4.5 a	58.9 ± 5.8 a	0.7 ± 0.5 a	0.7 ± 0.4 a


	n^veg^	0.58 ± 0.07 a	72.4 ± 14.0 b	2.6 ± 0.5 a	5.6 ± 0.3 b	16.1 ± 6.9 b	2.7 ± 0.9 a	0.9 ± 0.5 a


*Post-silking N partitioning was measured via the ^*15*^N method. Within reproductive treatments, vegetative treatment means for each parameter followed by different letters (a–b) are significantly different at *p* < 0.05 (*n* = 4). Vegetative treatments means are always compared within reproductive treatments.*

### Yield, Yield Components and Leaf Performance During Grain-Fill

Under post-silking N and water stress, yield was consistently greater in N^veg^ relative to n^veg^ (Table 4), indicating that the pre-silking luxury N reserve buffered yield against N and/or water stress. However, under adequate post-silking N and water supply (NW^rep^), the luxury N reserve did not offer any benefit in terms of yield (Table 4). The yield components driving the differences in grain yield between N^veg^ and n^veg^ under post-silking stress was kernel number, not kernel weight (Table 4).

**Table 4:** Effect of the vegetative treatments (N^veg^ and n^veg^) on yield, yield components and total biomass (mean ± S.E.) across all four reproductive treatments (NW^rep^, nW^rep^, Nw^rep^, nw^rep^) at maturity.

Reproductive Treatments	Vegetative Treatments	Grain yield (g plant^-1^)	Kernel number per plant	Kernel weight (mg kernel^-1^)	Total biomass at maturity (g plant^-1^)
High N + Well watered (NW^rep^)	N^veg^	133.0 ± 8.9 a	575 ± 30 a	231.2 ± 7.7 a	277.2 ± 15.7 a
	n^veg^	129.7 ± 12.9 a	532 ± 54 a	247.0 ± 12.1 a	291.6 ± 14.2 a
Low N + Well watered (nW^rep^)	N^veg^	139.2 ± 5.5 a	615 ± 17 a	226.0 ± 5.4 a	281.8 ± 12.3 a
	n^veg^	107.4 ± 8.9 b	512 ± 49 b	214.5 ± 7.5 a	224.6 ± 18.8 b
High N + Water Stress (Nw^rep^)	N^veg^	107.5 ± 6.3 a	548 ± 18 a	194.4 ± 11.9 a	229.5 ± 15.3 a
	n^veg^	78.1 ± 9.2 b	401 ± 51 b	203.4 ± 6.3 a	213.1 ± 12.4 a
Low N + Water Stress (nw^rep^)	N^veg^	91.3 ± 6.4 a	500 ± 43 a	192.7 ± 15.7 a	222.8 ± 9.9 a
	n^veg^	66.6 ± 4.9 b	371 ± 30 b	182.6 ± 9.2 a	169.9 ± 11.7 b


*Within reproductive treatments, vegetative treatment means for each parameter followed by different letters (a–b) are significantly different at *p* < 0.05 (*n* = 6). Vegetative treatment means are always compared within reproductive treatments.*

**FIGURE 2:**
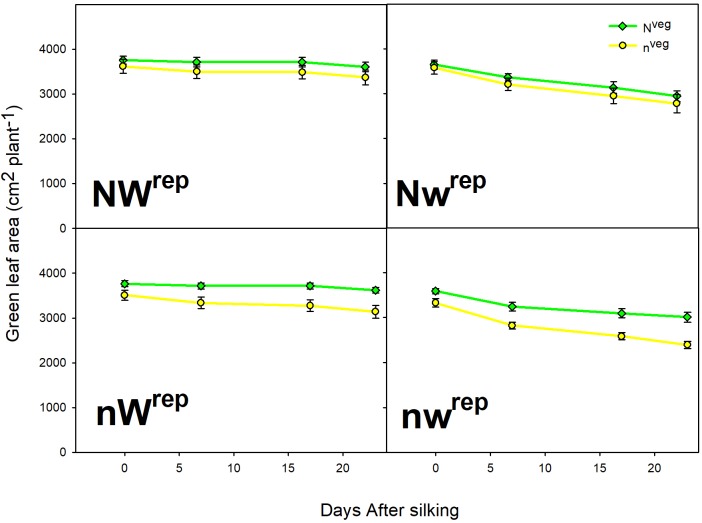
Green leaf area from silking to mid grain-filling in both vegetative treatments within all four reproductive treatments (mean ± S.E.). Repeated measures analysis indicates that the rate of green leaf area decline was significantly lower in N^veg^ compared to n^veg^ in the nW^rep^ and nw^rep^ treatments at *p* < 0.05 (*n* = 6). No significant differences were found in the rate of leaf area decline between N^veg^ and n^veg^ in the NW^rep^ and Nw^rep^ treatments at *p* < 0.05 (*n* = 6).

**FIGURE 3:**
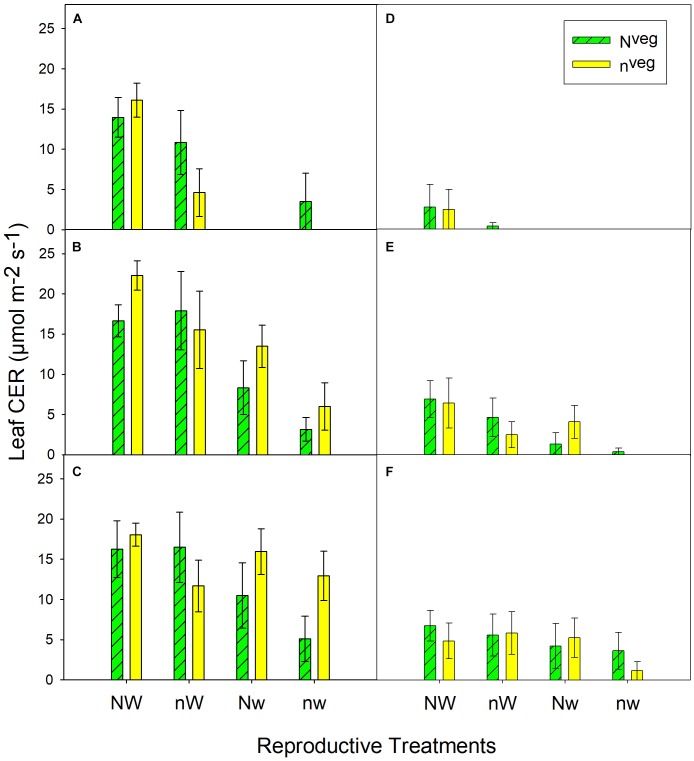
Leaf CER measured at mid grain-fill (21 days after silking; **A–C**) and late grain-fill (34 days after silking; **D–F**) on the 2nd leaf below the ear **(A,D)**, the ear leaf **(B,E)** and the 2nd leaf above the ear **(C,F)** (mean ± S.E.). Vegetative treatment means nested within reproductive treatments were statistically similar at both dates and at all 3 leaf positions at *p* < 0.05 (*n* = 6). Missing bars indicate that leaves were fully senesced at that leaf position.

During early and mid grain-fill, from 0 to 23 days after silking (DAS)^1^, leaf area was maintained longer in N^veg^ compared to n^veg^. This difference was statistically significant under post-silking N stress (nW^rep^ and nw^rep^), indicating that the reserve of luxury pre-silking N could buffer leaf function under N stress, though this effect was not significant under post-silking water stress (Figure 2). Leaf CER was statistically similar across N^veg^ and n^veg^ at mid grain-fill (21 DAS) and late grain-fill (35 DAS) on all three leaf positions (Figure 3). Thus, despite plants in the N^veg^ treatment remobilizing more than twice as much pre-silking N during grain-fill (Table 2), neither leaf area nor leaf CER was negatively affected compared to n^veg^ during grain-fill. By maturity total biomass was greater in N^veg^ when post-silking N stress occurred (Table 4). This suggests that the improvements in post-silking leaf function observed in N^veg^ under post-silking N stress translated into greater post-silking assimilate production and biomass accumulation.

## Discussion

This study reveals the mechanisms by which luxury N uptake accumulated before silking can buffer yield against N and/or water stresses experienced during reproductive growth. With adequate N and water supply imposed five days after silking (NW^rep^), kernel number and yield were similar between N^veg^ and n^veg^, indicating that luxury N did not benefit yield in non-stress conditions. When post-silking N and water were limiting growth factors, yield and kernel abortion were buffered by the presence of luxury N accumulated in the N^veg^ treatment (Table 4). At the same time, ^15^N tracers revealed that plants in N^veg^ remobilized more pre-silking N to the grain under post-silking N and/or water stress and remobilized N comprised an increasingly larger fraction of grain N compared to n^veg^ (Table 2). Notably, pre-silking luxury N uptake was associated with greater maintenance of green leaf area during early and mid grain-fill under post-silking N stress (nW^rep^ and nw^rep^) (Figure 2), and no reduction in leaf CER (Figure 3) or post-silking N uptake (Table 3) was observed in any reproductive treatment. These data suggest that vegetative N reserves accumulated in the N^veg^ plants buffered yield by preventing kernel abortion due to: (i) greater N remobilization, (ii) enhanced assimilate production during grain-fill.

Kernel number is particularly sensitive to N stress during the first ∼20 days after silking (Jacobs and Pearson, 1991; Below, 1997; Monneveux et al., 2005), when about 25% of total grain N is accumulated (Ning et al., 2017). The stalk is relied on heavily as a source of remobilized N (Cliquet et al., 1990; Chen et al., 2015; Yang et al., 2016), particularly during the first week or two after silking (Crawford et al., 1982; Ta and Weiland, 1992; Ning et al., 2017). Because the N and/or water stress treatments were imposed five days after silking, it is reasonable to believe that in the N^veg^ treatment, the reserve of stalk N was remobilized to the grain, preventing abortion of pollinated kernels after silking and improving kernel utilization of assimilates. At silking, stalk N content of N^veg^ plants was much greater than n^veg^ (1.0 vs. 0.5 g N plant^-1^; Figure 1) while stalk biomass was similar (88 g plant^-1^ vs. 84 g plant^-1^). It appears that the stalk N reservoir can be remobilized under stress to prevent shortfalls in grain N supply. In NW^rep^, when post-silking N uptake was high relative to the three other post-silking treatments, final kernel number was similar in N^veg^ and n^veg^, indicating that this reserve was not required when post-silking N supply was adequate. Because leaf area, leaf CER and total biomass at silking were equivalent between N^veg^ and n^veg^, it appears unlikely that shortfalls in assimilate production around silking reduced kernel number in n^veg^. Rather, the shortfall in grain N supply may have reduced the ability of pollinated kernels to utilize assimilates. Investigating the reasons for decreased yield in N deficient plants, Ning et al. (2018) found that from silking to 20 days after silking, N deficiency reduced the ability of developing kernels to utilize assimilates, leading to feedback inhibition of photosynthesis (i.e., starch accumulation in the ear leaf). The luxury N accumulated in N^veg^ may have enabled better utilization of assimilates by the developing kernels. A more rigorous challenge to this theory using time-course experiments, with frequent sampling in the 2 weeks following silking, is needed. Yet luxury N reserve may not be as effective in terms of buffering yield if water stress disrupts kernel set at silking. For example, Schussler and Westgate (1994) found that plants with greater N reserves had similar levels of kernel abortion during a 2-day water stress imposed at pollination. In a companion experiment (unpublished), we found that when kernel set was reduced due to vegetative water stress, luxury N uptake did not protect against yield loss.

Importantly, we find that the much larger amount of pre-silking N remobilized in the N^veg^ treatment did not impinge on physiological processes related to yield. Green leaf area in N^veg^ from early to mid grain-fill was equal to or greater compared to n^veg^, which led to greater biomass accumulation in the N^veg^ treatments under post-silking N stress. Within each post-silking treatment, both leaf CER and post-silking N uptake were similar across N^veg^ and n^veg^ treatments, indicating that much greater rates of N remobilization were maintained in N^veg^ without the typically observed reduction in leaf function or post-silking N uptake that typically occurs when N remobilization is enhanced by N stress. It is also notable that despite differences in grain yield (Table 4), post-silking N uptake was similar between N^veg^ and n^veg^ under reproductive stress (Table 3). Several studies have found that grain yield is a major driver of post-silking N uptake (Akintoye et al., 1999; Worku et al., 2007; Liu et al., 2014), yet data from this experiment suggests that other factors, such as post-silking N availability or pre-silking N uptake, are important regulators of post-silking N uptake. We found that at all three nodal positions on both post-silking sampling dates, leaf CER was consistently greater in NW^rep^ and Nw^rep^ compared to nW^rep^ and nw^rep^ (Figure 3), indicating that post-silking N supply may increase assimilate supply, although this difference in leaf CER was numeric only. Leaf senescence was enhanced by post-silking water stress, irrespective of N supply, similar to the findings of Wolfe et al. (1988). But in conditions where water stress occurs concomitantly with N stress, it appears that accumulating a reserve of vegetative N can reduce the magnitude of the negative effect of water stress on leaf senescence. Mean kernel weight was reduced under combined water/N stress (nw^rep^) relative to NW^rep^ (231 mg kernel^-1^ vs. 182 mg kernel^-1^; Table 4). Because kernel weight reductions occur when post-silking source-sink ratio declines (Borrás and Gambín, 2010; Hisse et al., 2019), it appears that N^veg^ was unable to completely prevent deterioration in the source-sink ratio during grain-fill under combined post-silking N and water stress.

Surprisingly, spikelet count at silking was affected by the vegetative N treatments (Table 1). Research from field settings research suggests that only severe N stress can reduce spikelet establishment at silking (Lemcoff and Loomis, 1986, 1994; DeBruin et al., 2018). A potential explanation for these results is that in the n^veg^ treatment spikelets were either smaller, or kernel rows were less uniform toward the tip of the ear. Small spikelets and uneven spikelet rows would have reduced the number of spikelets counted at silking. In this experiment, final kernel number was always much less than spikelet number at silking, particularly under N and/or water stress when the proportion of aborted spikelets was 31 and 35% for N^veg^ and n^veg^, respectively. This is similar to the findings of Yan et al. (2018), who found that at high planting densities, up to 26% of fertilized kernels can be aborted after fertilization. But even in the NW^rep^ treatment, the proportion of aborted spikelets reached 29% in N^veg^ and 19% in n^veg^. The magnitude of kernel number reductions under adequate N and water supply (NW^rep^) suggests that spikelet number at silking was not a major determinant of final kernel number, since yield was the same in the NW^rep^ treatment. Most studies conclude that spikelet number is not generally a major determinant of final kernel number given that more spikelets are produced than survive to maturity even in non-stress conditions (Tollenaar, 1977; Vega et al., 2001; DeBruin et al., 2018). However, it is possible that by increasing spikelet production, luxury pre-silking N uptake allows for greater plasticity in the response of final kernel number to stress. A larger amount of spikelets at silking would presumably buffer final kernel number if abiotic stress would cause a fixed percentage of kernels to abort.

Several authors have noted the antagonistic relationship between the remobilization of N accumulated during vegetative growth and post-silking N uptake (Gallais et al., 2007; Ciampitti and Vyn, 2013). Mechanistically, this trade-off is thought to be a function of competition for N and assimilates between the grain and the roots (Triboi and Triboi-Blondel, 2002). Root system function declines after silking as deposition of N and carbohydrates in kernels reduces assimilate supply to the roots and enhances N remobilization from the roots (Osaki, 1995; Liedgens et al., 2000; Yu et al., 2014). This intra-plant competition for assimilates is enhanced by N remobilization from leaves, which reduces both overall assimilate production and assimilate allocation to roots (Pan et al., 1995; Rajcan and Tollenaar, 1999). In this experiment, while N remobilization was twice as high in N^veg^ compared to n^veg^, there was no difference in post-silking N uptake. This may be because, in N^veg^, green leaf area was preserved despite much greater N remobilization. In a recent meta-analysis conducted by Mueller and Vyn (2016), there was surprisingly no evidence of a trade-off between remobilized and post-silking N. The authors speculated that this finding was due to the prevalence of studies in their dataset which used stay-green hybrids, known to maintain green leaf area by reducing leaf N remobilization in the face of strong grain N demand (Antonietta et al., 2014). Our findings provide support for the assertion that if a sufficiently sized reserve of pre-silking N is available, N remobilization that would otherwise reduce source strength is avoided. Kosgey et al. (2013) similarly speculated that utilization of stalk N in maize can delay leaf senescence during reproductive growth. We also found that under post-silking N stress, N^veg^ plants partitioned a greater fraction of N uptake to vegetative organs, which may have helped preserve organ function despite large amounts of N remobilization. Luxury N reserves accumulated pre-silking appear to resolve the “yield dilemma” in maize by allowing N remobilization to proceed without undue reductions in source strength, uncoupling N remobilization from reductions in post-silking N uptake.

Field studies find that in the absence of severe stress, N remobilization comprises between 35 and 65% of grain N (Ta and Weiland, 1992; Gallais et al., 2007; Ciampitti and Vyn, 2012; de Oliveira Silva et al., 2017). Under severe stress, however, this proportion shifts considerably. In NW^rep^, N remobilization comprised 53% of grain N, a value comparable to other studies. But under any post-silking N and/or water stress, N remobilization comprised a much greater proportion of grain N, particularly in N^veg^ plants with a reserve of luxury pre-silking N. This latter finding is comparable to those of other studies which imposed severe post-silking N stress. In a greenhouse study, when N was completely withheld after silking, 100% of grain N was derived from remobilized N (Friedrich and Schrader, 1979). In the field under less contrived conditions, Ta and Weiland (1992) also found that N stress increases the proportion of grain N derived from N remobilization; in one hybrid remobilized N comprised 70% of grain N under N stress. The amount of pre-silking N remobilized to the grain is largely a function of sink strength (i.e., grain yield) (Ciampitti and Vyn, 2013), so while N remobilization is enhanced under N stress, pre-silking N stress severe enough to decreases sink strength would presumably counteract this effect. In the present study, N stress was imposed five days after silking, and the high sink strength in N^veg^ may have stimulated greater N remobilization. Another explanation for the greater N remobilization observed in this study is the amount of pre-silking N uptake in the N^veg^ treatment. In the greenhouse, maize can take up substantial amounts of N if large quantities are provided (e.g., Subedi and Ma, 2005); pre-silking N uptake in greenhouse conditions may be greater than what is typically achieved in field conditions, permitting larger amounts of N to be remobilized.

We overcame a major complication associated with using ^15^N isotope tracers to quantify N remobilization. By growing plants in calcined clay, it was possible to leach ^15^N–KNO_3_ using irrigation, allowing for ^15^N applied during vegetative growth to be removed quickly and prior to reproductive growth. Our findings show that this method eliminates ^15^N–KNO_3_ in the pots and hence the chance that ^15^N will be taken up outside of the intended labelling period. This method can be useful in other ^15^N approaches and pulse-chase experiments, particularly in those circumstances when hydroponic experiments are not suitable, such as when imposing drought stress or otherwise trying to better replicate field conditions.

Unlike field-based ^15^N studies which quantify pathways of ^15^N fluxes in the field (e.g., Portela et al., 2006; O’Brien et al., 2012; de Oliveira Silva et al., 2017), this study was focused on the partitioning of ^15^N across maize organs (i.e., once assimilated into the plant). Both newly acquired N and previously acquired N are constantly cycling within the maize plant as proteins turn over (Gallais et al., 2006). Yet in this experiment, partitioning of post-silking N was measured only at maturity, reflecting the final destination of N at the end of the plant life cycle. The present study found that at maturity the leaves were very weak sinks for post-silking N, while stalks and roots were strong post-silking N sinks. However, ^15^N partitioning measured at maturity does not necessarily reflect the organs into which post-silking N was previously incorporated. de Oliveira Silva et al. (2017) measured post-silking N uptake in-season from silking to maturity. They identified much greater allocation of post-silking N to leaves (25–42%) during early and mid grain-fill, and that post-silking N allocation to leaves declined under N stress. In another ^15^N time-course study, Ta and Weiland (1992) found that while stalk N decreases from V14 to ∼30 days after silking as stalk N is remobilized to the ear, during the latter half of grain-fill stalk N content increases. By maturity, about 10–15% of ^15^N was found in the leaves depending on hybrid and N supply (Ta and Weiland, 1992). In a separate field study, Yang et al. (2017) found that leaf N content declined from silking to maturity while stalk N content increased. The low values of post-silking N allocated to the leaves in this experiment (less than 5%), and the large values of post-silking N allocated to the stalk and roots, are likely an artifact of the single sampling time at maturity, by which time large amounts of leaf N were already remobilized to other organs, and thus does not reflect in-season N allocation. Time-course measurements of post-silking N uptake are required to assess which organs post-silking N is assimilated into during grain-fill. It is, however, striking that vegetative organs such as the stalk and roots can be major sinks of post-silking N at maturity, and that under certain conditions, only a relatively small fraction of post-silking N uptake is partitioned to the grain.

While extending results from a greenhouse study to the field can only be done with caution, the results of this experiment have important implications for N fertilizer use in maize. Contemporary approaches to N fertilizer management are based on increasing the synchrony between crop N demand and N supply (Cassman et al., 2002; Chen et al., 2011). Maize N demand is typically conceptualized as a function of biomass accumulation rates, and so once crop growth rates are known, one can apply precisely the amount of N required to maintain that crop growth (Cassman et al., 2002; Sadras and Lemaire, 2014). The results of this study point to limitations in N fertilizer management strategies that either conceptualize maize N demand as a function of crop growth rates or take point estimates of crop N sufficiency/deficiency at vegetative stages of development (e.g., remotely sensed canopy reflectance; Pfeffer et al., 2010), since the potential importance of excessive or luxury N uptake in the face of future N stress cannot be accounted for. In years where high N losses are suspected due to weather, or where drought conditions limit post-silking N accessibility, proactive N management that provides for a degree of luxury pre-silking N uptake may be beneficial. Measures of crop N uptake at silking based on a sufficiency index or canopy LAI and leaf N content (Sadras and Lemaire, 2014), may be helpful in assessing luxury N uptake in maize. We also found that maize yield responded positively to additional post-silking N (N^veg^ to NW^rep^), suggesting that reactive N management up to R1 may be beneficial. Validation of these conclusions requires further exploration under field conditions, since it is unclear how results from this greenhouse experiment translate to production settings where maize is grown. However, at the level of the individual maize plant, this study demonstrates that luxury N accumulated prior to silking buffers yield under post-silking N and/or water stress.

## Data Availability

The datasets generated for this study are available on request to the corresponding author.

## Author Contributions

JN conceived the project, led the design of the experiment, data analysis and manuscript writing. HE and BD assisted with project development, experimental design and manuscript writing.

## Conflict of Interest Statement

The authors declare that the research was conducted in the absence of any commercial or financial relationships that could be construed as a potential conflict of interest.

## Footnotes

1 Harvest occurred 39 DAS (Oct 1).
