# Supplementary material for: Luxury Vegetative Nitrogen Uptake in Maize Buffers Grain Yield Under Post-silking Water and Nitrogen Stress: A Mechanistic Understanding
Source: Front Plant Sci. 2019 Mar 26;10:318. doi: 10.3389/fpls.2019.00318 (PMC6443847; doi:10.3389/fpls.2019.00318)
Supplement: Supplementary file 1 [file Table_1.DOCX]

**Table S1** Total N content (nitrate-N + ammonium-N) in Turface^®^ remaining after irrigating buckets for 30 minutes (18 L min^-1^). 2.25 g N was added to each pot initially.

| Pot Number | Proportion of N remaining after irrigation (%) |
| --- | --- |
| 1 | 1.2 |
| 2 | 0.9 |
| 3 | 1.1 |
| 4 | 0.2 |

**S1. Regression leaf area from a leaf are meter on: a) ruler measurements of leaf length and width, b) leaf dry weight**

At silking, on a subset of 350 leaves, leaf length and width at the widest point was measured with a ruler before being detached from the plant. The 350 leaves were then measured using a leaf area meter (LI-3100c, LI-COR, Lincoln, NE). This allowed for a regression of leaf area as measured by the leaf area meter against leaf length and width as measured by a ruler. Leaves in this subset were dried in an oven at 80^o^C for 48 hours and weighed individually to parameterize another regression equation expressing leaf area (as measured by the leaf area meter) as a function of dried leaf weight. This regression was used to calculate senesced leaf area during reproductive growth. The R^2^ of the regression of leaf area (ruler) to leaf area (leaf area meter) (n=350) was equal to 0.96:

Leaf area (cm^2^) = -57.03 + 6.05*leaf_width + 1.63*leaf_length +0 .61*(leaf_length*leaf_width)

For the regression of leaf area (leaf area meter) to leaf weight, the two vegetative treatments required separate regression equations:

N^veg^ senesced leaf area (cm^2^) = 144.00*senesced_leaf_weight + 38.62 (R^2^ = 0.91)

n^veg^ senesced leaf area (cm^2^) = 148.49*senesced_leaf_weight + 40.14 (R^2^ = 0.92)

**Table S2** δ^15^N (‰) of maize plant tissue in pots where 40 mg ^15^N (as KNO_3_^-^ at 10% atom excess) was previously added and then leached away before seeding by irrigating for 30 minutes at a flow rate of 18L min^-1^. The negative δ^15^N (‰) illustrates that the irrigation procedure prevented subsequently grown plants from being labelled with ^15^N above natural abundance^[[1]](#footnote-1)^.

| Pot Number | δ^15^N value of sampled plant tissue reported against the primary reference scale of atmospheric air (‰) |
| --- | --- |
| 1 | -1.59 |
| 2 | -2.29 |
| 3 | -2.03 |
| 4 | -3.88 |

**Fig S1** Leaf CER (µmol m^2^ s^-1^) of the vegetative treatments (N^veg^ and n^veg^) at silking on leaves at three nodal positions (mean ± S.E). Vegetative treatments are compared within leaf nodal position. Different letters indicate significant differences in leaf CER across N^veg^ and n^veg^ treatments at *p* < 0.05 (n=6).

**Fig S2** Proportion of vegetative N remobilized to the grain at maturity across all eight treatments (mean ± S.E). Vegetative treatments are nested within the reproductive treatments. Different letters indicate significant differences between N^veg^ and n^veg^ treatments at *p* < 0.05 (n=4).

1. The δ^15^N (‰) of the sample was calculated following Mariotti et al. (1981): δ^15^N (‰) = ((R_sample_ / R_air_ -1) × 1000 [↑](#footnote-ref-1)
